# Self-Care by Muslim Women during Ramadan Fasting to Protect Nutritional and Cardiovascular Health

**DOI:** 10.3390/ijerph182312393

**Published:** 2021-11-25

**Authors:** Marta López-Bueno, Ángel Fernández-Aparicio, Emilio González-Jiménez, Miguel Ángel Montero-Alonso, Jacqueline Schmidt-RioValle

**Affiliations:** 1 Department of Nursing, Faculty of Health Sciences, University of Granada, 52071 Melilla, Spain; martalopez@ugr.es; 2 Department of Nursing, Faculty of Health Sciences, University of Granada, Av. Ilustración, 60, 18016 Granada, Spain; anfeapa@ugr.es (Á.F.-A.); jschmidt@ugr.es (J.S.-R.); 3 Department of Statistics, O.I. Faculty of Medicine, University of Granada, Av. Investigación, 11, 18016 Granada, Spain; mmontero@ugr.es

**Keywords:** Ramadan, obesity, bioimpedance, anthropometry, eating habits

## Abstract

The practice of Ramadan involves a series of changes in lifestyle, mainly in eating habits. The research aim of this study is to determine the prevalence of overweight-obesity, the degree of compliance with dietary recommendations and the effects of religious fasting on cardiovascular health among a population of Muslim women living in Melilla, a Spanish city in North Africa. A follow-up cohort study was conducted on 62 healthy adult women (33.6 ± 12.7 years). Anthropometric and body composition parameters were obtained using bioimpedance and dietary records. All of the participants were overweight or obese, especially due to the non-compliance with dietary recommendations; however, more than 60% considered their weight was appropriate or even low. By the end of Ramadan, the women’s body mass index and fat component values had fallen significantly (*p* < 0.001), but this loss was later recovered. Dietary records revealed an excessive consumption of lipids and sodium, and the presence of a high waist-to-hip ratio. All of these factors are related to cardiovascular risk. In conclusion, promoting nutritional health and encouraging year-round self-care among adult Muslim women is necessary in order to ensure healthy fasting during Ramadan.

## 1. Introduction

During the month of Ramadan, Muslims are required to abstain from smoking, sexual intercourse, eating and drinking, from dawn until nightfall. The average duration of the daytime fast depends on the date and the place of residence [1], since the Muslim calendar follows the lunar system and each month moves through the solar seasons. Some researchers consider this practice a model of intermittent fasting, characterised by the sudden introduction of modifications in dietary habits [2]. In general, Muslims have two meals during the night, one when the fast is broken, shortly after sunset, and the other just before sunrise. This change in eating schedules also affects other activities of daily life [3].

The effects of Ramadan on nutritional health have been widely studied in countries with a Muslim majority, but few such studies have been undertaken with respect to women in a Western context, although in these settings, too, it is women who are mainly responsible for family care [4]. Moreover, women are usually more vulnerable to certain pathologies, especially those related to nutrition [5].

For many years, obesity has been a major problem worldwide, even in European countries where the Mediterranean diet is deeply rooted. For example, in Spain, levels of obesity have risen throughout the population, but especially among adult women [6].

In view of these considerations, together with forecasts of continuing rises in overweight-obesity [7] and taking into account the known association between visceral adipose tissue and cardiovascular health [8], the present community-bases nursing study was undertaken to determine the prevalence of overweight and obesity in a group of Muslim women living in a Western society. The study consists of an analysis of nutritional habits, of compliance with recommendations for micronutrient and macronutrient intake, and of whether the observance of Ramadan strictures is associated with variables related to cardiovascular risk.

## 2. Materials and Methods

### 2.1. Study Design and Sampling

**Figure 1:**
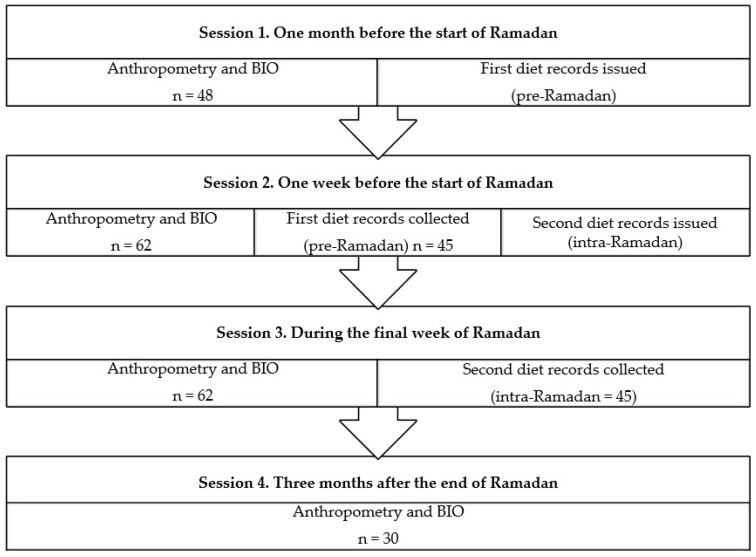
Study sessions. BIO: Bioimpedance.

This follow-up cohort study was conducted on a female Muslim population resident in Melilla, a Spanish city on the coast of North Africa. The sample initially consisted of 62 women, aged 18–61 years. All participants were Muslim women, of legal age, who voluntarily observed the Ramadan precepts. Women with a chronic or acute disease, or who were pregnant or had a metal prosthesis were excluded. Figure 1 shows the flow chart for the sessions conducted.

### 2.2. Data Collection

In accordance with the calendar for Ramadan during the study period, the first session took place one month before the start of the fast. The second session was held one week before the start of Ramadan, the third in the last week of Ramadan and the final one, three months after Ramadan had ended. In Session 1, each participant was given a dietary record to be completed before the start of Ramadan, and anthropometric and body composition parameters were measured and recorded. In Session 2, these dietary questionnaires were collected, those to be completed during Ramadan were handed out, and the second measurement and recording of anthropometric and body composition parameters was performed. This process was repeated in Session 3, and in the final session, the last measurement and recording of anthropometric and body composition parameters was carried out. Of the 62 women who were given the dietary record to be completed during the corresponding moments of the study, only 45 delivered the two records completed in full. Thus, the loss to study was 27.4%. However, there is no evidence that the loss of these data was related to the nutritional status of the participants. We suggest that this reluctance to participate might have arisen from the study design and/or the characteristics of the religious precepts studied.

This study was conducted in full accordance with the guidelines and ethical principles for medical research in human beings established by the World Medical Association in the Declaration of Helsinki (Finland, 1994), and as reviewed in successive assemblies, the most recent of which was the 64th General Assembly, held in Fortaleza (Brazil) in October 2013. All participants were informed in detail about the study goals and characteristics, and gave signed informed consent to take part. Furthermore, in accordance with the General Regulations on Data Protection and with Organic Law 3/2018, of 5 December, on the protection of personal data and digital rights, data confidentiality was assured; the participants’ anonymity was protected by the use of codes. Moreover, these data were used solely for the scientific purposes presented.

### 2.3. Anthropometric Evaluation and Body Composition Analysis

Anthropometric variables were measured following the protocol of the International Society for the Advancement of Kinanthropometry [9]. All variables were measured at the same time of day, between 9 a.m. and 12 noon, and by the same person. The participants were asked to attend without having eaten beforehand, wearing comfortable clothes, with no metal objects in direct contact with the skin, with an empty urinary bladder and without having practiced intense physical exercise in the previous twelve hours. The following anthropometric variables were studied: total body weight (BW), height, body mass index (BMI), waist circumference (WC), waist/hip ratio (W/Hp) and waist/height ratio (W/Ht). Waist and hip circumferences were measured with a flexible, non-extensible Seca^®^ tape measure, with an accuracy of 1 mm. The waist circumference was measured at the midpoint between the lower rib margin and the iliac crest, with the abdomen relaxed. The hip circumference was measured at the level of maximum circumference of the buttocks. Each perimeter was measured in triplicate by the same observer, and the mean value was taken. The W/Hp ratio was calculated by dividing the waist circumference (cm) by the hip circumference (cm). For this ratio, a score <0.8 was considered within the range of normal values. The W/Ht ratio was calculated by dividing the waist circumference (cm) by the height (cm). For this ratio, a score of 0.4–0.5 was considered normal.

Height was measured using a portable TANITA^®^ stadiometer, with the participant standing upright, with her head oriented according to the Frankfort plane and with the trunk and pelvis in continuous contact with the vertical branch of the stadiometer. The horizontal branch was then applied to the top of the head. From these weight and height measurements, the BMI was calculated to categorise the participant as normoweight, overweight or obese in accordance with the criteria established by the World Health Organisation (WHO) [10].

In addition to the above, a body composition study was conducted using a TANITA^®^ SC-330 body composition analyser, which is self-calibrating and has an accuracy of ±100 g. This apparatus was also used for weight estimation. The following body composition variables were determined: percentage of fat mass, total fat mass, lean mass, muscle mass, percentage of water content, total water content, visceral fat and bone mass.

### 2.4. Food Consumption

Dietary intake was determined using a 72-h dietary record, in which the participants noted the types and amounts of food consumed, and the procedure used for food preparation. To maximise the precision of the quantities recorded, each participant was given an information sheet with equivalents and other practical indications for use in the home. 

The dietary recommendations proposed by the Spanish Society of Community Nutrition (SENC) in 2011 [11] and the WHO in 2008 [12] were used to assess the compliance with recommendations for micronutrient and macronutrient intake of Muslim women in each session.

### 2.5. Statistical Analysis

All statistical analyses were performed using IBM SPSS software v.24 (SPSS Inc., Chicago, IL, USA). For the descriptive analysis, the mean and standard deviation were calculated for each quantitative variable, while the qualitative variables were described by proportions. All variables were assessed in each of the four study sessions, which were compared by a one-way ANOVA. Student’s *t*-test (with Levene’s test for the equality of variances) was applied for the variables that presented a normal distribution, and, otherwise, the Wilcoxon test was used. The normality of the distributions was verified using the Shapiro-Wilk test.

The effect of fasting during Ramadan (Sessions 2 and 3) was observed using the Wilcoxon test for paired samples. As some values were highly dispersed, an analysis of the medians of the variables was also conducted, before and during Ramadan. Finally, to determine the possible influence of fasting on cardiovascular risk, an analysis of odds ratios, with 95% confidence intervals, was carried out, using a logistic regression model adjusted for the present age and for earliest age on starting observance of Ramadan. In every case, the level of significance assumed was *p* < 0.05.

## 3. Results

### 3.1. Sociodemographic Characteristics

Table 1 shows that slightly over half of the participants were single, while nearly 34% were married (33.9%); the women’s mean age was 33.6 ± 12.7 years. In terms of education background, the sample was divided into the women who had completed basic, secondary or higher education and also those who had no formal education.

### 3.2. Anthropometric Measurements and Bioimpedance

Table 2 shows that the mean BMI values obtained, at each of the moments studied, represent overweight or obese. However, the value recorded for Session 2 was significantly higher than the recorded for Session 3 (*p* < 0.001). Moreover, it can be observed a decrease of obesity prevalence and an increase of overweight prevalence in Session 3, in comparison to Session 2. Similar differences between these two moments were observed in the mean values obtained by bioimpedance for the percentage of fat mass (*p* < 0.001) and visceral fat (*p* = 0.002).

Among the anthropometric values related to central adiposity, the only significant differences found were for hip circumference (*p* = 0.008), between Sessions 2 and Session 3. Notably, the mean values for waist circumference and W/Hp were above normal values at all times during the study.

### 3.3. Dietary Intake: Macronutrients and Micronutrients

Table 3 shows that some macronutrients, such as vitamin D and calcium, do not undergo significant changes during fasting (*p* = 0.843 and *p* = 0.200, respectively). However, a significant increase was observed in the intake of phosphorus (*p* = 0.001), cholesterol (*p* < 0.001), trans-fatty acids (*p* = 0.002), vegetable fibre (*p* < 0.001) and all macronutrients.

**Figure 2:**
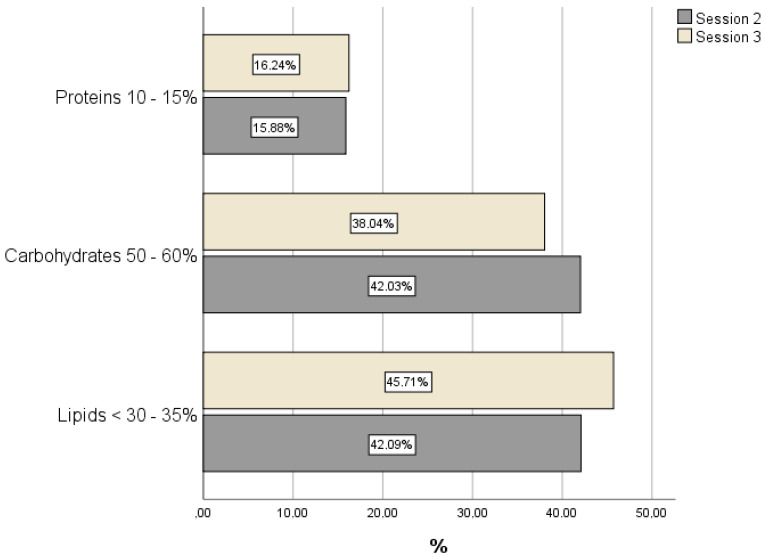
Contribution of macronutrients to total caloric intake during Sessions 2 and 3.

Figure 2 illustrates the imbalance in the caloric and lipid profiles recorded, before and during the final week of Ramadan.

### 3.4. Cardiovascular Risk during Ramadan

Table 4 shows the influence of the fasting state (before and during Ramadan) on the variables related to cardiovascular risk. The variables related to central adiposity most strongly associated with the observance of Ramadan were W/Hp ratio and visceral fat, with an adjusted OR of 1.62 and of 1.61 respectively. Regarding the variables derived from the dietary record, the change in fasting status only affected potassium (OR 2.10, 95% CI [1.01, 4.35]), which became abnormally elevated during Ramadan. By contrast, below-normal values were obtained during Ramadan for monounsaturated fatty acids (OR 0.36, 95% CI [0.17, 0.78]) and total cholesterol (OR 0.16, 95% CI [0.06, 0.39]).

## 4. Discussion

The BMI is the most widely-used parameter to define and classify people as overweight and obese. However, body composition techniques provide a more realistic approximation of these concepts [13]. Accordingly, the present study also includes bioimpedance analysis. In addition, measures of central adiposity are obtained, to evaluate the possible effects of Ramadan on cardiovascular health.

Our results show that BMI values fell during Ramadan, which corroborates previous research in this area, conducted in different countries [14,15,16,17]. The prevalence of obese women during the final week of Ramadan also fell, which is in consonance with the decrease of the percentage of body fat and visceral fat also observed during the final week of Ramadan. Despite this slight decrease of obesity prevalence, the values of W/Hp and W/Ht did not suffer any change, and the levels were above the normal values. In relation to the changes in body fat that are associated with fasting, some authors have affirmed that a loss of body weight is always accompanied by a corresponding reduction in body fat. Thus, in a study carried out in Sanliurfa (Turkey), a significant reduction was observed in the percentage of body fat and visceral fat, according to bioimpedance results and the waist/hip ratio [18]. Another investigation, in Indonesia, also measured a loss of body fat. In this case, however, the reduced W/Hp at the end of the fasting period was not significant [19]. In the present investigation, a significant decrease was observed in the percentage of body fat and visceral fat obtained by bioimpedance, but not in the anthropometric measurement of central adiposity. Moreover, unlike the above-mentioned studies, in our participants the W/Hp and W/Ht ratios were always above the recommended values.

In addition to dietary factors, the participants’ smoking and physical activity habits were considered. In this respect, none of the women in our study were smokers, and all claimed to have maintained the same level of physical activity during Ramadan as in the rest of the year. The dietary records obtained in Sessions 2 and 3 revealed a certain imbalance in the intake of micronutrients and macronutrients. In relation to the intake of micronutrients, a notable finding is the deficit of two vitamins that are essential for adults (according to a study of nutritional objectives for the Spanish population) [11], namely folic acid and vitamin D. Although by the end of Ramadan mean levels of folic acid had increased significantly, the dietary record obtained in Session 2 indicates a daily intake below the recommended 400 μg/day for women aged 14–70 years [11,12]. Similarly, the intake of vitamin D was well below the recommended 10 µg/day throughout the study [11,12]. In this respect, the increasing presence of obesity in the population, the use of sunscreen and the decreased consumption of dairy foods all tend to provoke vitamin D deficiency, despite the many hours of sunshine received in this part of Spain [20].

Regarding the minerals related to bone health, the participants’ dietary intake of phosphorus was high, both in Session 2 and in Session 3. Significantly, a high intake of phosphorus and a low intake of calcium are both risk factors for inadequate bone mineralisation. For this reason, it is normally recommended to limit the consumption of processed foods, which are the main source of inorganic phosphates [21]. In addition, hyperphosphataemia is reported to have a negative impact on cardiovascular health [22].

Another mineral that presented levels of dietary intake higher than the dietary recommendations [11,12], especially during Ramadan, was potassium. This may be due to the substitution of salt by potassium chloride, which is a widespread practice in the preparation of processed foods, as a means of preventing arterial hypertension. However, high levels of sodium intake were also recorded in Sessions 2 and 3. The excessive consumption of salt (sodium chloride), in addition to its widely known prejudicial effects, favours microvascular endothelial inflammation, anatomical remodelling and other functional abnormalities, even in persons with normal blood pressure [23].

Regarding the intake of macronutrients, the dietary records reveal an imbalance in the caloric profile in Sessions 2 and 3, with lipids constituting the main source of energy, to the detriment of carbohydrates, especially during the final week of Ramadan. These data differ significantly from the recommendations by the SENC [11]. In our study, the intake of vegetable fibre was significantly augmented during Ramadan, and the levels were above the dietary recommendations [11,12]. The decreased intake of total cholesterol recorded at the end of Ramadan is a positive trend. Nevertheless, the mean values recorded remained above the recommended level [11,12]. These findings are consistent with previous dietary research carried out in Spain, such as the ENIDE study [24] and the Food Consumption Panel [25]. Similar results were reported by the ANIBES study, which recorded a dietary imbalance in women of all age groups, but especially among older women [5]. However, in none of these previous studies was the participants’ religion taken into consideration. On the other hand, and despite the influence that religion may exert on nutritional habits, a relevant consideration is that the Muslim women in our study reside in a Western European context.

The study results we report highlight the presence of various cardiovascular risk factors among the participants, chiefly overweight/obesity and high waist/hip and waist/height ratios, throughout the study period. Moreover, the weight loss observed during Ramadan is not a protective factor, since this loss is fully recovered three months later. These findings are in line with previous reports, according to which the average weight loss during Ramadan is just over one kilogram, which is subsequently recovered or even increased [26,27]. Another study concluded that Ramadan observance had a neutral effect on health, in view of the ephemeral nature of the changes recorded [28]. In addition, we report an association among the observance of Ramadan and the presence of elevated W/Hp ratio and visceral fat, variables which have been shown to be associated with cardiometabolic risk factors [29]. These results are not in agreement with those of Silveira et al. [30] and Yang et al. [31], who reported that intermittent fasting had positive effects on reducing cardiovascular risk factors in obese women. These differences are probably due to the non-compliance with dietary recommendations by the participants of our study, which have been previously discussed. 

Regarding the dietary habits observed, only the increased potassium intake represents a special health risk during Ramadan. However, it is unknown whether the elevated potassium levels associated with increased dietary intake have adverse effects among the general population since, to date, the only studies conducted in this respect have focused on adults with normal renal function [32]. Nevertheless, our findings regarding the dietary habits of the participants corroborate the generalised trend away from the Mediterranean diet in many European countries, despite the evidence of its importance in preventing cardiovascular risks [33].

A relevant consideration is the geographic context of the city of Melilla, which is subject to the cultural influence of neighbouring countries in the Middle East and North Africa. In these regions, it is socially acceptable, or even desirable, for women to be overweight [34]. The possible influence of Arab culture might be apparent in the fact that more than half of the women in our study perceived themselves as having normal weight, despite the objectively high values recorded.

In summary, all of the participants were overweight or obese. Although their BMI values decreased during Ramadan, they had returned to baseline three months later. At no stage of the study did the participants comply with the recommendations for the intake of macronutrients and micronutrients, but the imbalance was heightened during Ramadan. For this reason, it is necessary to ensure healthy fasting during Ramadan by promoting a balanced intake of macronutrients and micronutrients. 

Among the strengths of our study, to our knowledge it is pioneering in the sense that no previous investigation has been made of the dietary impact of fasting, as a feature of religious observance by Muslim women before, during and after Ramadan. Among its limitations, it was not possible to analyse the participants’ lipid profile during Ramadan, as almost all of them stated that drawing a blood sample might invalidate their fast. Another limitation was that the sample size of our study is low. 

## 5. Conclusions

The BMI values of our participants decreased during Ramadan, but they had returned to baseline three months later. Moreover, the participants did not comply with the recommendations for the intake of macronutrients and micronutrients throughout the study, with a higher imbalance during Ramadan. Despite the association observed between the observance of Ramadan and the visceral fat and W/Hp ratio, it cannot be concluded that Ramadan observance has a direct impact on cardiovascular health. To clarify these questions, further study is necessary, in a Western European context, such as the one we describe, including, in addition, Muslim women of normal weight. For the present, however, we consider it necessary to put into practice policies and actions aimed at promoting nutritional health and encouraging year-round self-care among adult Muslim women, since only the establishment of appropriate lifestyle habits will ensure healthy fasting during the observance of religious precepts. 

## Figures and Tables

**Table 1:** Sociodemographic characteristics.

Variables	Mean ± SD
Age	33.6 ± 12.7
Number of children (*n*)	1 ± 1.5
Age at first Ramadan	12.3 ± 1.8
Marital status *	
Single	35 (56.5)
Married	21 (33.9)
Widowed	2 (3.2)
Divorced	4 (6.5)
Education *	
No formal education	8 (12.9)
Basic or elementary	23 (37.1)
Secondary	16 (25.8)
Higher education	15 (24.2)
Self-perception of present weight *	
Low	4 (6.5)
Normal	37 (59.7)
Overweight	19 (30.6)
Obese	2 (3.2)
Unknown	1 (1.6)

* Data are expressed as *n* (%).

**Table 2:** Anthropometric measurements and bioimpedance.

Variables	Session 1	Session 2	Session 3	Session 4	*p* *	*p* **
Weight (kg)	67.3 ± 12.6	67.2 ± 14.1	66.1 ± 14.0	68.3 ± 13.5	0.894	<0.001
BMI (kg/m^2^)	26.8 ± 6.4	26.3 ± 5.8	25.8 ± 5.6	26.4 ± 5.5	0.865	<0.001
Normoweight	24 (50.0)	29 (46.8)	29 (46.8)	14 (46.7)		
Overweight	9 (18.8)	18 (29.0)	21 (33.9)	9 (30.0)		
Obesity	15 (31.2)	15 (24.2)	12 (19.3)	7 (23.3)		
Waist CF (cm)	90.1 ± 11.9	90.1 ± 12.4	89.4 ± 12.4	90.3 ± 12.7	0.987	0.106
Hip CF (cm)	100.0 ± 12.5	99.0 ± 12.7	98.1 ± 12.7	100.0 ± 13.1	0.850	0.008
W/Hp	0.9 ± 0.0	0.9 ± 0.0	0.9 ± 0.0	0.9 ± 0.0	0.361	0.633
W/Ht	0.5 ± 0.1	0.5 ± 0.1	0.5 ± 0.1	0.5 ± 0.1	0.967	0.104
% Fat mass	32.6 ± 9.0	32.1 ± 9.4	31.4 ± 9.5	33.2 ± 9.4	0.822	<0.001
Fat mass (kg)	23.0 ± 9.8	22.8 ± 10.7	22.0 ± 10.5	23.7 ± 10.3	0.886	<0.001
Lean mass (kg)	44.4 ± 3.9	44.7 ± 4.1	44.3 ± 4.05	44.6 ± 4.0	0.989	0.259
Muscle mass (kg)	42.1 ± 3.6	42.2 ± 3.9	42.0 ± 3.8	42.3 ± 3.8	0.991	0.286
Total water (kg)	31.3 ± 3.1	31.3 ± 3.4	31.1 ± 3.3	31.4 ± 3.3	0.980	0.182
% Water	47.4 ± 5.8	47.7 ± 6.0	47.6 ± 6.2	47.0 ± 6.0	0.960	0.897
Visceral fat	5.1 ± 4.0	5.0 ± 4.0	4.8 ± 3.8	5.1 ± 3.7	0.985	0.002
Bone mass (kg)	2.3 ± 0.2	2.3 ± 0.2	2.2 ± 0.2	2.3 ± 0.2	0.967	0.088

Data are expressed as mean ± SD or *n* (%). BMI: Body mass index; CF: Circumference; W/Hp: Waist/Hip ratio; W/Ht: Waist/Height ratio. Session 1: one month before Ramadan; Session 2: one week before Ramadan; Session 3: last week of Ramadan; Session 4: three months after Ramadan. * *p*-value. Session 1 vs. Session 4. ** *p*-value. Session 2 vs. Session 3.

**Table 3:** Dietary intake before Ramadan (Session 2) and immediately afterwards (Session 3).

Variables	Session 2	Session 3	*p*	Session 2	Session 3	*p*
	Mean ± SD	Mean ± SD		Median	Median	
Energy (kcal)	2292.8 ± 1576.0	3044 ± 1951.6	0.002	1880	2409.2	0.002
Carbohydrates (g)	240.9 ± 157.2	289.6 ± 127.2	0.002	224.0	257.0	0.002
Protein (g)	91.0 ± 63.42	123.3 ± 89.5	0.002	71.6	97.8	0.002
Lipids (g)	107.2 ± 93.1	154.7 ± 135.8	0.008	77.5	110.0	0.008
SFA (g)	31.3 ± 23.40	38.8 ± 24.76	0.075	25.7	31.6	0.075
MFA (g)	52.0 ± 51.16	80.6 ± 91.02	0.028	36.2	50.8	0.028
PFA (g)	14.0 ± 13.43	22.5 ± 14.73	<0.001	10.0	19.7	<0.001
Cholesterol (mg)	504.8 ± 969.01	658.3 ± 584.0	<0.001	247.0	548.0	<0.001
Trans-fatty acids	1.1 ± 1.2	1.6 ± 0.8	0.002	0.93	17,921.0	0.002
Vitamin B2 (mg)	2.4 ± 2.1	2.5 ± 1.9	0.223	1.8	1.9	0.223
Niacin (mg)	19.6 ± 16.4	27.5 ± 22.3	0.048	15.5	23.1	0.048
Vitamin B6 (mg)	2.7 ± 2.1	4.2 ± 3.3	0.001	1.9	3.1	0.001
Folic acid (µg)	317.1 ± 263.7	545.0 ± 593.73	<0.001	243.0	358.0	<0.001
Vitamin D (µg)	7.3 ± 10.9	5.4 ± 5.5	0.843	3.2	3.2	0.843
Vitamin E (mg)	11.6 ± 9.3	16.4 ± 14.5	0.012	8.8	11.0	0.012
Iron (mg)	18.8 ± 15.2	26.5 ± 18.7	0.001	12.9	20.2	0.001
Iodine (µg)	98.6 ± 81.2	130.0 ± 105.2	0.025	75.5	96.9	0.025
Calcium (mg)	928.2 ± 813.6	1017.3 ± 624.5	0.200	748.0	829.0	0.200
Magnesium (mg)	300.3 ± 165.9	449.3 ± 293.4	<0.001	260.0	359,0	<0.001
Sodium (mg)	2239.2 ± 1949.1	3162.1 ± 6386.7	0.731	1684.0	1864.0	0.731
Potassium (mg)	3189.1 ± 1657.8	5800.4 ± 4829.2	<0.001	2795.0	4377.0	<0.001
Phosphorus (mg)	1501.3 ± 1059.8	2049.5 ± 1273.6	0.001	1259.0	1642.0	0.001
Vegetable fibre (g)	23.2 ± 15.8	43.8 ± 32.0	<0.001	19.7	34.5	<0.001

SFA: Saturated fatty acids, MFA: Monounsaturated fatty acids, PFA: Polyunsaturated fatty acids.

**Table 4:** The association between the observance of Ramadan and variables related to cardiovascular risk.

Variable	*n*	%	OR	95% CI	OR ^a^	95% CI
Waist circumference (cm)						
Normal	19	48.7	1			
Elevated	43	50.6	1.08	0.50; 2.30	1.11	0.46; 2.67
W/Hp						
Normal	4	40.0	1			
Elevated	58	50.9	1.55	0.42; 5.80	1.62	0.41; 6.41
Fat mass (%)						
Normal	19	48.7	1			
Elevated	43	50.6	1.08	0.50; 2.30	1.08	0.50; 2.37
Visceral fat						
Normal	41	48.2	1			
Elevated	21	53.8	1.25	0.59; 2.68	1.61	0.53; 4.89
Sodium (mg)						
Normal	31	44.9	1			
Elevated	31	56.4	1.58	0.78; 3.23	1.59	0.78; 3.24
Potassium (mg)						
Normal	31	42.5	1			
Elevated	31	60.8	2.10 *	1.01; 4.35	2.14 *	1.02; 4.47
Saturated fatty acids (g)						
Normal	5	71.4	1			
Elevated	57	48.7	0.38	0.07; 2.04	0.37	0.07; 2.03
Monounsaturated fatty acids (g)						
Normal	29	65.9	1			
Elevated	33	41.3	0.36 *	0.17; 0.78	0.36 *	0.17; 0.78
Polyunsaturated fatty acids (g)						
Normal	0	0				
Elevated	62	50.0				
Total cholesterol (mg)						
Normal	28	80.0	1			
Elevated	34	38.2	0.16 **	0.06; 0.39	0.15 **	0.06; 0.38

The comparison variables are the Session 2 and 3. Data are presented as odds ratios (OR) with 95% confidence intervals (CI) using a logistic regression model. OR ^a^ adjusted for present age and age at first Ramadan. W/Hp: Waist-to-hip ratio. * *p* < 0.05; ** *p* < 0.001.

## Data Availability

The data presented in this study are available on request from the corresponding author.
